# Transcriptional and neurotransmitter signatures of cerebral spontaneous neural activity in nurses with burnout

**DOI:** 10.3389/fpubh.2025.1630294

**Published:** 2025-08-21

**Authors:** Chun-Mei Song, Jian-Ping Liu, Hu-Cheng Yang, Qing-He Li, Shu Wang, Hai-Juan Chen, Shu-Fang Wang, Li Chen, Si-Yu Gu, Feng Zhang, Ping-Lei Pan

**Affiliations:** ^1^School of Nursing and Rehabilitation, Nantong University, Nantong, China; ^2^Department of Disinfection Supply Center, Affiliated Hospital 6 of Nantong University, Yancheng Third People’s Hospital, Yancheng, China; ^3^Department of Neurology, Affiliated Hospital 6 of Nantong University, Yancheng Third People’s Hospital, Yancheng, China; ^4^Department of Radiology, Binhai Maternal and Child Health Hospital, Yancheng, China; ^5^Department of Nursing, Affiliated Hospital 6 of Nantong University, Yancheng Third People’s Hospital, Yancheng, China; ^6^Department of Radiology, Affiliated Hospital 6 of Nantong University, Yancheng Third People’s Hospital, Yancheng, China

**Keywords:** burnout syndrome, nurse, fractional amplitude of low-frequency fluctuations, imaging transcriptomics, neurotransmitter systems, precuneus

## Abstract

**Objective:**

To investigate the neural and molecular correlates of occupational burnout in nurses by integrating resting-state fMRI (rs-fMRI), clinical assessments, brain-wide gene expression, and neurotransmitter atlases.

**Methods:**

Fifty-one female nurses meeting burnout criteria and 51 matched healthy controls underwent 3 T rs-fMRI. We analyzed fractional amplitude of low-frequency fluctuations (fALFF) and seed-based functional connectivity (FC), correlating findings with burnout (emotional exhaustion [EE], depersonalization [DP], and personal accomplishment [PA]). The fALFF *t*-map was spatially correlated with Allen Human Brain Atlas gene expression (followed by gene ontology enrichment) and neurotransmitter system maps.

**Results:**

Nurses with burnout exhibited significantly decreased precuneus fALFF and reduced precuneus-right dorsolateral prefrontal cortex (DLPFC) FC compared to controls. The fALFF in the precuneus negatively correlated with EE and DP, and positively correlated with PA, while reduced precuneus-DLPFC FC negatively correlated with EE. Genes spatially associated with fALFF alterations were enriched in pathways involving neuronal excitability, synaptic organization, stress response, and immune modulation. The fALFF alteration pattern also spatially correlated with serotonin, norepinephrine, *γ*-aminobutyric acid, glutamate, and endocannabinoid system distributions.

**Conclusion:**

Nurse burnout features precuneus hypoactivity and precuneus-DLPFC hypoconnectivity, linked to EE and DP severity. Associated molecular signatures implicate altered neuronal excitability, stress/immune pathways, and multiple neurotransmitter systems. The precuneus-DLPFC circuit and identified molecular pathways represent potential targets for interventions against burnout.

## Introduction

Burnout syndrome is recognized as an occupational phenomenon resulting from chronic, unmanaged workplace stress, and is typically characterized by three core dimensions: emotional exhaustion (EE), depersonalization (DP) or cynicism, and reduced personal accomplishment (PA) (1–3). This syndrome represents a significant occupational hazard, particularly within high-stress professions such as nursing (4), where prevalence rates are notably high. A recent meta-analysis estimated the global prevalence of burnout among nurses over the last decade at 30%, with evidence suggesting an increasing trend (5). The consequences of nursing burnout extend beyond the individual’s well-being, manifesting as symptoms such as fatigue, cognitive weariness, depression, and poor sleep (1, 4, 5), to negatively impact patient safety, the quality of care, and overall healthcare system stability through increased nurse turnover and medical errors (4, 5). Given its profound impact, understanding the biological underpinnings of burnout in this critical workforce is crucial for informing effective prevention and intervention strategies (6).

Despite the significant clinical and societal burden imposed by burnout, particularly among nurses, its underlying neurobiological mechanisms remain incompletely understood (6, 7). Previous neuroimaging studies have begun to associate burnout with alterations in brain structure, observing changes in gray matter volume or cortical thickness in regions such as the prefrontal cortex and insula (8, 9). Functionally, studies have reported abnormal brain activity during tasks (10), disruptions in resting-state functional connectivity (FC), functional network topology, and functional connectome hierarchy (11–14). These findings suggest that burnout may involve structural/functional dysregulation across multiple levels of brain organization. Beyond examining how regions communicate (connectivity), understanding the baseline level of spontaneous activity within individual regions is also crucial. The amplitude of low-frequency fluctuations (ALFF) measures the intensity of local resting-state BOLD signals, reflecting spontaneous neuronal activity. Fractional ALFF (fALFF), a normalized version, improves upon ALFF by enhancing both sensitivity and specificity for detecting this regional spontaneous brain activity in resting-state fMRI (rs-fMRI) (15, 16). Although recent studies have begun to explore the relationship between fALFF and stress or shift work in nurses (17, 18), a key gap in current research lies in understanding the potential molecular mechanisms underlying these alterations in local spontaneous brain activity.

To address these knowledge gaps, this study employed a multimodal integrative analysis strategy. We utilized fALFF based on rs-fMRI to non-invasively probe spontaneous neural activity (15). Based on brain regions showing significant fALFF alterations, we further employed seed-based FC analysis to investigate whether the functional integration patterns between these core regions and other brain areas were also altered. To explore the clinical relevance of these neuroimaging findings, we examined the relationships between fALFF and FC alterations and the severity of core burnout symptoms in nurses. Crucially, to uncover the potential molecular underpinnings of burnout-related fALFF changes, we adopted an imaging transcriptomics approach, correlating the spatial maps of our observed fALFF alterations with genome-wide transcriptional expression data provided by the Allen Human Brain Atlas (AHBA) (19, 20). Furthermore, we extended our analysis by correlating the spatial maps of fALFF alterations with published brain maps of various neurotransmitter receptors and transporters (e.g., for serotonin [5-HT], norepinephrine, dopamine, *γ*-aminobutyric acid [GABA], glutamate, and endocannabinoid systems), to explore whether specific neurotransmitter systems are spatially associated with the observed local activity changes (21, 22). This integrative approach, combining neuroimaging, clinical assessments, and multi-level molecular atlases (gene transcriptome and neurotransmitter systems), holds the potential to provide a more comprehensive perspective on the neurobiological mechanisms of burnout in nurses.

## Methods

### Participants

Female nurses were recruited from the Affiliated Hospital 6 of Nantong University as participants in this study. Data collection took place from September 2024 to January 2025. The inclusion criteria for the burnout group were as follows: (1) females, aged 20–40 years; (2) right-handed; (3) meeting the criterion on at least one of the three dimensions of the Maslach Burnout Inventory-Human Services Survey (MBI-HSS) scale (EE ≥ 27 points, DP ≥ 8 points, and PA ≤ 24 points) (23), whereas the inclusion criteria for the healthy controls (HCs) were as follows: (1) females, aged 20–40 years; (2) right-handed; (3) scores below the critical values on all three dimensions (EE < 27 points, DP < 8 points, PA > 24 points). Individuals were excluded if they met any of the following criteria: (1) endocrine, neurological, or psychiatric disorders or other primary diseases; (2) current pregnant or lactating women; (3) history of drug dependence, smoking, or alcohol consumption; (4) adverse reactions during scanning leading to termination of the experiment or contraindications to MRI scanning; (5) data collection failure during scanning or poor image quality; (6) MRI images showing organic brain lesions; (7) other serious physical illnesses. Based on these criteria, a total of 102 right-handed participants were ultimately enrolled and assigned to two groups: a burnout group (*n* = 51) and an HC group (*n* = 51).

In this study, the Beck Anxiety Inventory (BAI) and Beck Depression Inventory (BDI) were used to measure the levels of anxiety and depression among all participants, respectively. The study was approved by the ethics committee of the Affiliated Hospital 6 of Nantong University (2024–82), and all participants provided written informed consent.

### Data acquisition and preprocessing

Resting-state fMRI data were acquired using a 3.0 T MRI scanner equipped with a 24-channel head coil (Discovery 750w, GE, United States) at the Affiliated Hospital 6 of Nantong University. During the fMRI scanning, participants were instructed to remain awake with their eyes closed, and to hold still as much as possible. Detailed fMRI parameters and preprocessing procedures are presented in the Supplementary material.

### fALFF and FC analysis

fALFF was calculated using the Data Processing Assistant for Resting-State fMRI (DPARSF) (24). The time series of each voxel was converted to the frequency domain using a fast Fourier transform (FFT). The square root of the power spectrum at each frequency was computed, and the average square root within the 0.01–0.1 Hz frequency band represents ALFF (16). fALFF was determined as the ratio of ALFF (0.01–0.1 Hz) to the power across the entire analyzed frequency range (0.01–0.25 Hz) (15). The fALFF values were standardized through Fisher’s z-transformation to enhance normality.

Seed-based FC was computed using DPARSF (24) by utilizing clusters that showed significant between-group differences in fALFF as seeds or regions of interest (ROIs). The mean time series of the ROI was extracted, and Pearson correlation coefficients were calculated between the ROI time series and the time series of all other brain voxels. Fisher’s z-transformation was applied to the resulting correlation maps for statistical analysis.

Between-group differences in fALFF and FC were assessed using a voxel-wise two-sample *t*-test, controlling for age, educational level, Total intracranial volume (TIV), BAI, and BDI scores as covariates. Statistical significance for both fALFF and FC analyses was determined at a voxel-level threshold of *p* < 0.001, with a cluster-level Family Wise Error (FWE) correction (*p* < 0.05) for multiple comparisons.

### Correlation analysis

Correlation analyses were conducted within the burnout group to examine the relationships between neuroimaging measures and burnout-related scores. Specifically, Pearson correlations were assessed between alterations in fALFF and FC and scores on the EE, DP, and PA subscales of the MBI.

### Brain gene expression data processing

Brain gene expression data were acquired from the AHBA dataset, which originated from six human postmortem donors (25, 26). The dataset includes the expression levels of over 20,000 genes across 3,702 spatially unique brain tissue samples, analyzed using custom 64 K Agilent microarrays. A previously established pipeline was employed to process gene expression data (27). Probe-to-gene annotations were revised using the Re-Annotator package, incorporating the latest data from the National Center for Biotechnology Information (28). Through intensity-based filtering, probes were excluded if they did not surpass the background noise in a minimum of 50% of samples across all donors. RNA-seq data were used to select probes for genes, excluding genes that were not common to both the RNA-seq and microarray datasets. Correlations between microarray and RNA-seq expression measures were calculated for the remaining genes, and probes with low correlations (*r* < 0.2) were eliminated. A representative probe for each gene was selected based on the highest correlation with RNA-seq data. Only left cerebral cortex tissue samples were included in the analysis due to all six donors having left hemisphere data, while right hemisphere samples were available for only two donors. Moreover, including subcortical samples could introduce biases because of significant gene expression differences between cortical and subcortical regions (26). To address inter-sample variations and donor-specific influences in gene expression, we applied within-sample cross-gene and within-gene cross-sample normalizations using the scaled robust sigmoid normalization method. Differential stability (DS) quantifies the consistency of regional expression variation across donor brains. Previous studies have indicated that genes exhibiting high DS exhibit more uniform spatial expression profiles among donors and are enriched in brain-related biological processes (25). To establish reliable transcriptome-neuroimaging spatial correlations, we selected genes with highly conserved expression patterns. Genes were ranked based on their DS values, and the top 50% were chosen for analysis. This resulted in normalized expression data for 5,013 genes across 1,290 tissue samples. We focused our analysis on tissue samples located within the Automated Anatomic Labeling (AAL) 90 atlas regions, resulting in a final sample × gene matrix of 838 × 5,013.

### Transcriptome-neuroimaging spatial correlation and gene category enrichment analysis

To assess the spatial correspondence between group differences in imaging metrics and gene expression, spherical ROIs with a radius of 3 mm were placed at the MNI coordinates of each of the 838 tissue samples. The average *t*-value of voxels within each spherical ROI was extracted from the group comparison statistical *t*-map for fALFF. Subsequently, Pearson correlations between the expression profile of each gene and the pattern of *t*-values across these 838 tissue sample locations were computed gene-wise, resulting in 5013 spatial correlation coefficients, denoted as gene scores. Following the approach by Fulcher et al. (29), neuroimaging-spatial ensemble-based gene category enrichment analysis (GCEA) was conducted for the gene scores as follows: First, updated gene ontology (GO) term hierarchy and annotation files were acquired from the Gene Ontology^1^ on March 16, 2025. Second, direct gene-to-category annotations were carried out for the 5,013 AHBA genes, focusing on GO categories with 10–200 annotations. Third, gene scores were aggregated at the GO category level as the mean score of genes annotated to each category. Fourth, 10,000 surrogate maps with spatial autocorrelation matching the original *t*-map were generated using the BrainSMASH package^2^ based on the spatial-lag model (30). Null distributions, known as the neuroimaging-spatial ensemble-based null model, were created for mean gene scores of each GO category by assessing spatial correlations between gene expression and the 10,000 spatial autocorrelation-preserving surrogate maps. Finally, the statistical significance of a GO category was determined by comparing its score from the actual data with the neuroimaging-spatial ensemble-based null. A two-sided *p*-value threshold of < 0.05 was set for significance assessment (i.e., above or below the null).

### Correlation with neurotransmitters

To investigate the potential relationship between burnout-related fALFF alterations and the spatial distribution of neurotransmitter systems, we utilized JuSpace,^3^ a tool for analyzing spatial correlations between multimodal neuroimaging data. We examined the spatial correlations between the group comparison fALFF *t*-map and published PET/SPECT-derived maps encompassing various neurotransmitter systems, including dopamine, serotonin, glutamate, GABA, acetylcholine, opioid, cannabinoid, noradrenaline, and fluorodopa. Pearson correlation coefficients were computed between the *t*-map and neurotransmitter maps across 90 AAL regions. This analysis adjusted for spatial autocorrelation and partial volume using the gray matter probability map. Significance was determined at *p* < 0.05, with exact *p* values calculated through spatial permutation-based null maps with 5,000 permutations.

## Results

### Demographic and clinical characteristics

The demographic and clinical characteristics of the burnout and HCs are presented in Table 1. There were no significant differences between the two groups in terms of age (Burnout group: median 34 years, Interquartile Range [IQR] 27–37; HC group: median 34 years, IQR 28–36), years of education, TIV, blood pressure, and body mass index (BMI) (all *p* > 0.05; see Table 1 for details). However, compared to the HCs, the burnout group exhibited significantly higher scores on the EE, DP, BAI, and BDI, and significantly lower scores on the PA (all *p* < 0.05; see Table 1 for details).

**Table 1:** Demographic information and clinical data.

Characteristics	Nurses with burnout	HCs	*P*
N	51	51	-
Age (years)	34 (27, 37)	34 (28, 36)	0.77^a^
Education (years)	16 (16, 16)	16 (16, 16)	0.98^a^
TIV (mm^3^)	1424.50 ± 134.52	1414.17 (1360.24, 1539.99)	0.20^a^
SBP (mmHg)	114.78 ± 8.25	116.20 ± 10.36	0.51^b^
DBP (mmHg)	70.43 ± 7.75	70 (65, 80)	0.37^a^
BMI	21.48 (19.63, 23.63)	20.94 (19.81, 24.01)	0.97^a^
EE	23.14 ± 11.94	13 (8, 17)	*< 0.001* ^a^
DP	12.25 ± 5.98	3 (0, 5)	*< 0.001* ^a^
PA	24.53 ± 9.66	39 (31, 43)	*< 0.001* ^a^
BAI	28 (25, 34)	24 (22, 26)	*< 0.001* ^a^
BDI-II	8 (4, 14)	3 (0, 7)	*< 0.001* ^a^

Data are represented as mean ± SD or median (quartiles). Italic represents a significant difference between two groups of people. HCs, healthy controls; TIV, intracranial total volume; SBP, systolic blood pressure; DBP, diastolic blood pressure; BMI, body mass index; EE, emotional exhaustion; DP, depersonalization; PA, reduced personal accomplishment; BAI, beck anxiety inventory; BDI-II, beck depression inventory-II.

a The Mann–Whitney U test was used to obtain the *p* values.

b The Student’s *t*-test was used to obtain the *p* values.

### fALFF differences between groups

**Figure 1:**
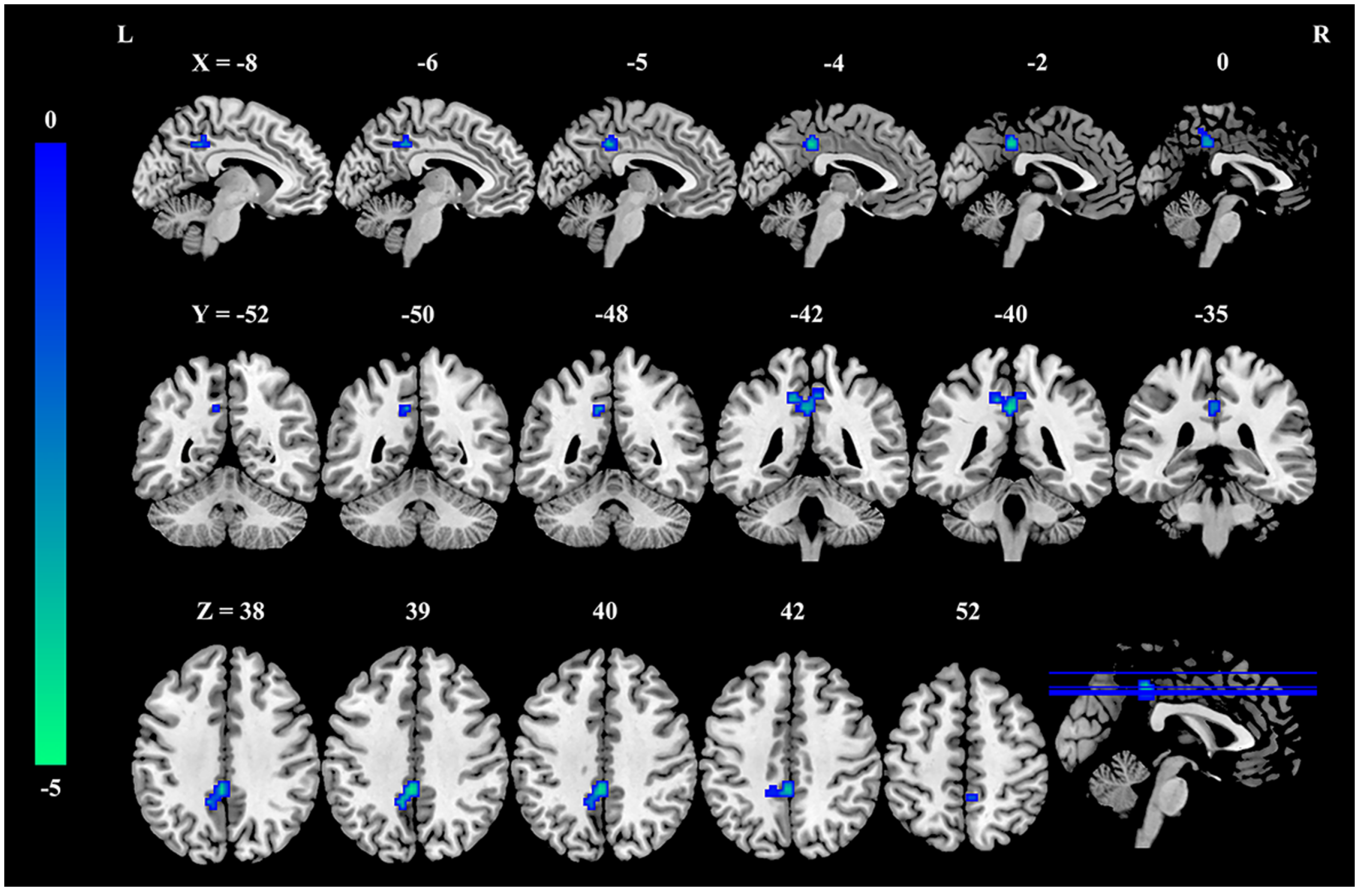
Decreased fALFF in the precuneus of nurses with burnout compared to healthy controls. Compared with healthy controls, fALFF in the precuneus was significantly decreased in the burnout group (voxel-wise *p* < 0.001, cluster-level family-wise error corrected *p* < 0.05). The color bar indicates *t*-values. fALFF, fractional amplitude of low-frequency fluctuations; L, left; R, right.

Compared to the HC group, the burnout group exhibited a significant reduction in fALFF values within a cluster in the precuneus (MNI coordinate: x = −15, y = −42, z = 45; cluster size = 38 voxels, *t* = −4.07, *P*_FWE-corrected_ < 0.05), as illustrated in Figure 1 and Table 2.

**Table 2:** Group differences in fALFF between nurses with burnout and HCs.

Regions (AAL)	Cluster size	MNI coordinate (mm)			*t*
		x	y	z	
PCUN	38	−15	−42	45	−4.07

Compared to HCs, nurses with burnout showed a significant decrease in fALFF in the precuneus. fALFF, fractional amplitude of low-frequency fluctuations; HCs, healthy controls; AAL, anatomical automatic labeling; PCUN, precuneus.

### FC differences between groups

**Figure 2:**
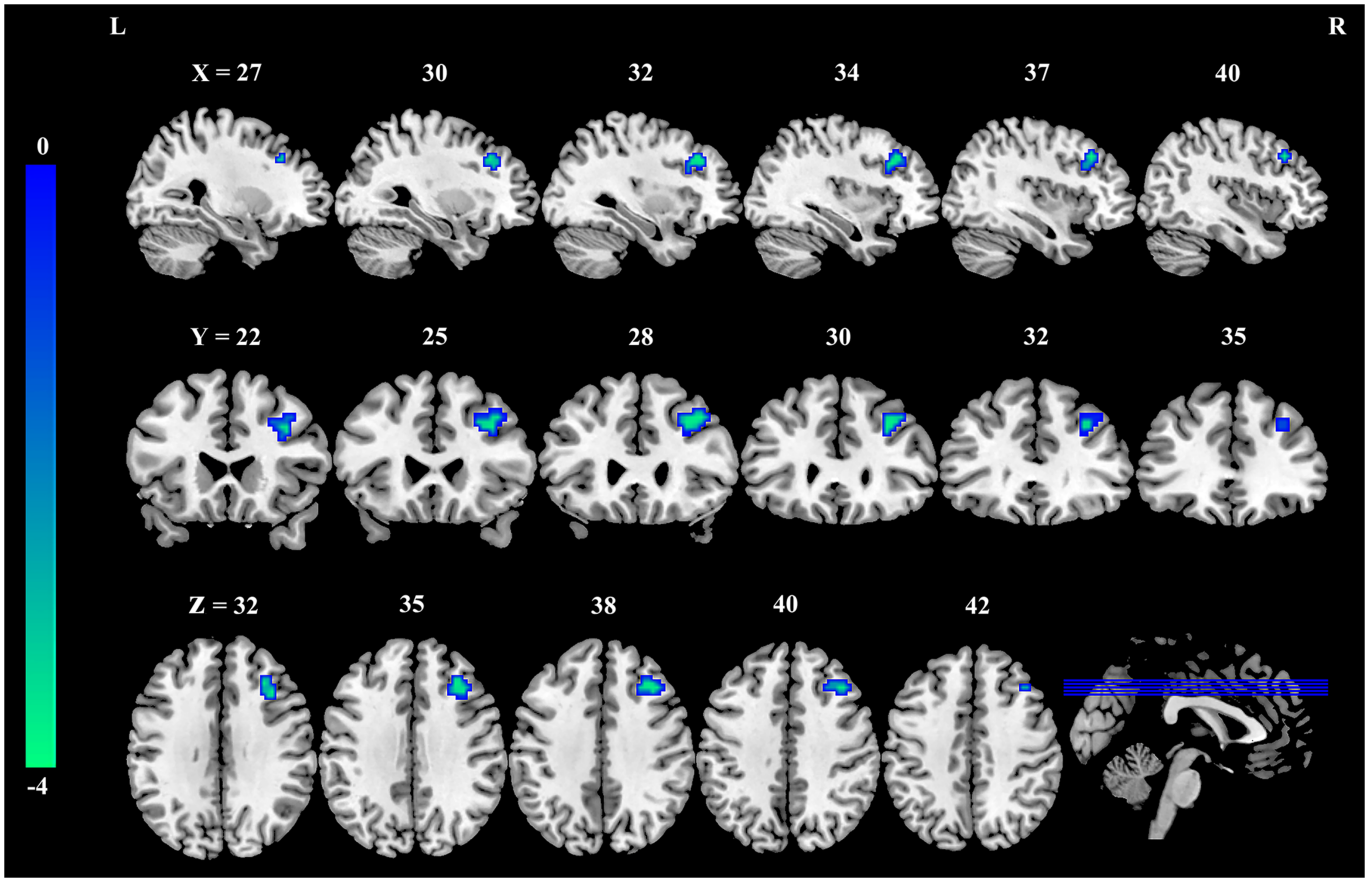
Reduced FC between the precuneus and right dorsolateral prefrontal cortex in nurses with burnout. Compared to healthy controls, the FC between the precuneus and the right dorsolateral prefrontal cortex was significantly decreased in the burnout group (voxel-wise *p* < 0.001, cluster-level family-wise error corrected *p* < 0.05). The color bar indicates *t*-values. FC, functional connectivity; L, left; R, right.

Seed-based FC analysis, utilizing the precuneus cluster identified above as the seed region, demonstrated a significant reduction in FC between the precuneus seed and a cluster in the right dorsolateral prefrontal cortex (DLPFC) in the burnout group compared to the HCs (MNI coordinate: x = 33, y = 21, z = 24; cluster size = 46 voxels, *t* = −4.02, *P*_FWE-corrected_ < 0.05, Figure 2, and Table 3).

**Table 3:** Group differences in FC between nurses with burnout and HCs.

Seed region	Regions (AAL)	Cluster size	MNI coordinate (mm)			*t*
			x	y	z	
PCUN	DLPFC.R	46	33	21	24	−4.02

Compared to HCs, FC between the PCUN and DLPFC.R is significantly reduced in nurses with burnout. FC, functional connectivity; HCs, healthy controls; PCUN, precuneus; AAL, anatomical automatic labeling; DLPFC, dorsolateral prefrontal cortex; R, right.

### Correlation analysis

Within the burnout group, correlation analysis revealed significant negative correlations between mean fALFF values extracted from the precuneus cluster and EE (*r* = −0.290, *p* = 0.039) and DP (*r* = −0.312, *p* = 0.026). A significant positive correlation was observed between precuneus fALFF values and PA (*r* = 0.287, *p* = 0.041). Furthermore, a significant negative correlation was found between mean FC values representing the precuneus-right DLPFC connection and EE (*r* = −0.308, *p* = 0.028) within the burnout group. Detailed results of these correlation analyses were available in the Supplementary Table S1.

### Gene categories spatially correlated with burnout fALFF alterations

**Figure 3:**
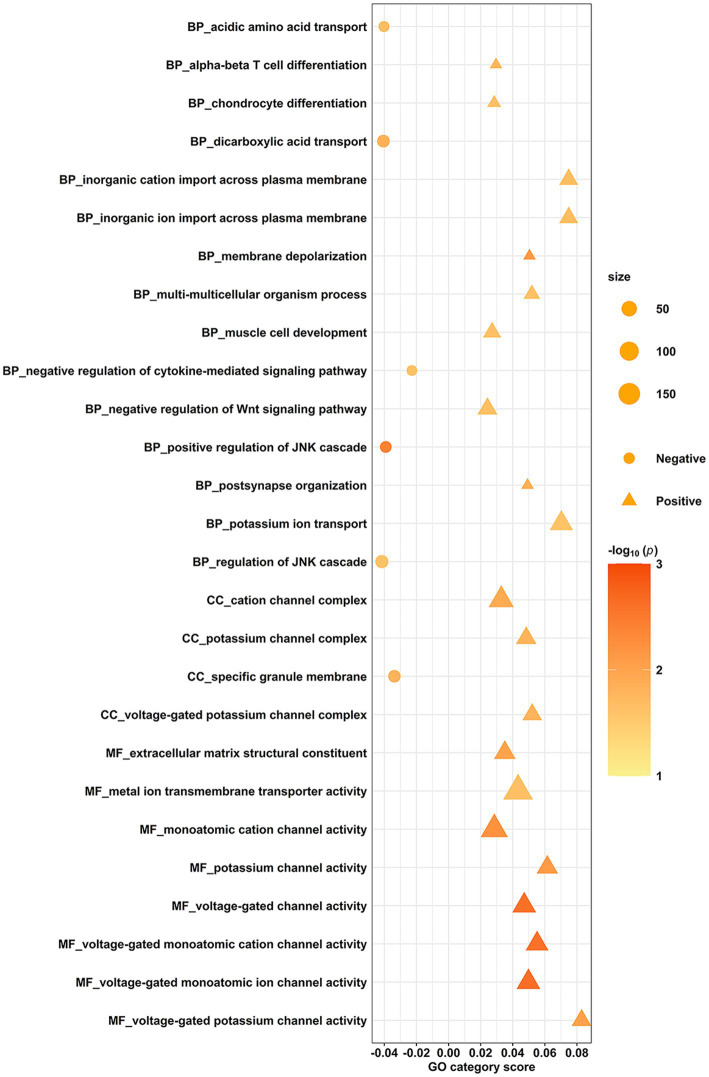
Gene categories associated with fALFF alterations of burnout. The spatial correlation between transcriptome and neuroimaging, along with ensemble-based GCEA, revealed that the fALFF correlates of burnout were linked to gene expression in GO categories related to membrane depolarization, cytokine-mediated signaling pathway, JNK, postsynapse organization, potassium ion, and voltage-gated channel. The y-axis indicates the GO category, while the x-axis represents the GO category score. The color signifies the spatially constrained permutation-based statistical significance of the spatial correlations, presented as -log10(*P*). Positive associations are denoted by triangles, and negative associations are denoted by circles. GCEA, gene category enrichment analysis; fALFF, fractional amplitude of low-frequency fluctuations; GO, gene ontology; JNK, c-Jun N-terminal kinase; Wnt, Wingless/Integrated; BP, biological process; CC, cellular component; MF, molecular function.

The spatial correlation between transcriptome-neuroimaging and the ensemble-based GCEA indicated that the fALFF alterations of burnout were linked to gene expression of GO categories mainly involving membrane depolarization, cytokine-mediated signaling pathway, c-Jun N-terminal kinase (JNK), postsynapse organization, potassium ion, and voltage-gated channel (spatially-constrained permutation-based *p* < 0.05). Specifically, the fALFF alterations in burnout were positively correlated with the alpha-beta T cell differentiation, chondrocyte differentiation, inorganic cation import across plasma membrane, inorganic ion import across plasma membrane, membrane depolarization, multi-multicellular organism process, muscle cell development, negative regulation of Wingless/Integrated (Wnt) signaling pathway, postsynapse organization, potassium ion transport, cation channel complex, potassium channel complex, voltage-gated potassium channel complex, extracellular matrix structural constituent, metal ion transmembrane transporter activity, monoatomic cation channel activity, potassium channel activity, voltage-gated channel activity, voltage-gated monoatomic cation channel activity, voltage-gated monoatomic ion channel activity, and voltage-gated potassium channel activity. Conversely, they were negatively correlated with positive regulation of JNK cascade, dicarboxylic acid transport, acidic amino acid transport, negative regulation of cytokine-mediated signaling pathway, regulation of JNK cascade, and specific granule membrane (See Figure 3 for full details).

### Neurotransmitters associated with burnout fALFF alterations

**Figure 4:**
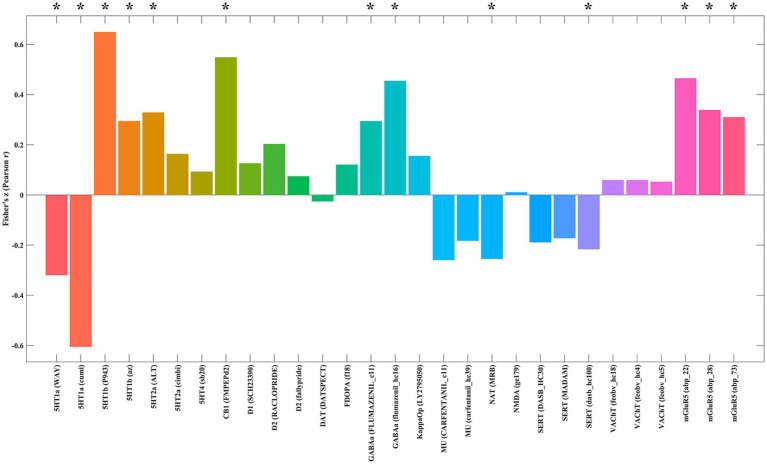
Spatial correlations between the fALFF alteration map and neurotransmitter system maps. Spatial correlation analyses showed that differences in fALFF between burnout and HCs were significantly negatively correlated with 5HT1a, NAT, and SERT, and significantly positively correlated with 5HT1b, 5HT2a, CB1, GABAa, and mGluR5. fALFF, fractional amplitude of low-frequency fluctuations; HCs, healthy controls; 5HT1a, 5-Hydroxytryptamine receptor 1a; NAT, Norepinephrine Transporter; SERT, Serotonin Transporter; 5HT1b, 5-Hydroxytryptamine receptor 1b; 5HT2a, 5-Hydroxytryptamine receptor 2a; CB1, Cannabinoid Receptor Type 1; GABAa, *γ*-Aminobutyric acid sub-type A receptors; mGluR5, metabotropic glutamate receptor 5.

Cross-regional spatial correlation analysis using JuSpace revealed significant correlations between the fALFF *t*-map and the spatial distribution maps of several neurotransmitter receptors and transporters (*p* < 0.05, permutation-corrected; Figure 4 and Supplementary Table S2). Significant positive correlations were found between *t*-values in the fALFF map and the density maps of 5-Hydroxytryptamine receptor 1b (5HT1b), 5-Hydroxytryptamine receptor 2a (5HT2a), Cannabinoid Receptor Type 1 (CB1), *γ*-aminobutyric acid sub-type A receptors (GABAa), and metabotropic glutamate receptor 5 (mGluR5). Significant negative correlations were observed between *t*-values and the density maps of 5HT1a, Norepinephrine Transporter (NAT), and Serotonin Transporter (SERT).

## Discussion

This study employed a multi-level approach integrating resting-state fMRI, clinical assessments, and spatial molecular atlas data to elucidate the neurobiological correlates of occupational burnout in female nurses. We identified significant alterations in spontaneous brain activity and FC in nurses experiencing burnout compared to HCs. Specifically, the burnout group exhibited decreased local spontaneous activity (fALFF) within the precuneus. Furthermore, FC analysis revealed decreased functional integration between the precuneus and the right DLPFC. Importantly, the magnitude of these neuroimaging alterations demonstrated clinical relevance, as precuneus fALFF negatively correlated with EE and DP scores, while positively correlating with PA scores. Additionally, the reduced precuneus-DLPFC connectivity was negatively associated with EE scores. Extending beyond functional alterations, our imaging transcriptomics analysis provided initial insights into the potential molecular underpinnings. Genes whose spatial expression patterns spatially covaried with the observed fALFF alterations were significantly enriched in pathways crucial for neuronal excitability (e.g., membrane depolarization, voltage-gated and potassium ion channels), synaptic organization, stress response (JNK pathway), and immune modulation. Complementing this, spatial correlation analyses indicated associations between the altered fALFF pattern and the distribution of key neurotransmitter systems, including specific serotonin (5HT1a, 1b, 2a, SERT), norepinephrine (NAT), GABA (GABAa), glutamate (mGluR5), and endocannabinoid (CB1) receptors/transporters. Taken together, these converging findings provide novel evidence for specific disruptions in local brain activity and FC communication in nurse burnout, link these disruptions to core clinical symptoms, and suggest potential underlying molecular mechanisms involving altered neuronal function, stress responses, neuroinflammation, and multiple neurotransmitter systems.

A central finding of this study is the significantly reduced spontaneous neural activity, indexed by fALFF, within the precuneus among nurses experiencing burnout. Specifically, lower fALFF activity in the precuneus corresponded to higher EE and DP scores, while higher fALFF activity in this region was linked to higher PA scores. The precuneus, a core hub of the default mode network (DMN), is critically involved in integrating self-referential processing, episodic memory retrieval, visuospatial imagery, consciousness, and the sense of the physical or bodily self (31). Notably, causal evidence highlights the precuneus’s role in maintaining body schema and self-association, with perturbations leading to dissociative experiences like depersonalization (32–34). Previous research consistently links structural and functional alterations in the precuneus and associated DMN regions to chronic stress, exhaustion disorder, burnout, and dissociative states (14, 32, 35–37). Specifically, chronic occupational stress has been associated with reduced FC within the posterior DMN, including the precuneus (14, 37). The observed hypoactivity (lower fALFF) in the precuneus in our burnout group may therefore reflect disruptions in introspection, self-awareness, the integration of personal experiences, or even a disturbed sense of bodily self. These processes are often reported as impaired in burnout and explicitly linked to depersonalization/dissociation (32–34, 36, 38–40). Intriguingly, a meta-analysis by Messina et al. found that the emotion regulation strategy of acceptance (a non-judgmental stance towards emotions) was specifically associated with decreased activity in the PCC/precuneus compared to control conditions (41). This raises the possibility that the reduced precuneus activity in burnout might reflect a maladaptive form of detachment or a dysfunctional attempt at emotional acceptance that contributes to the syndrome’s characteristic numbing and exhaustion.

Complementing this local finding, we identified decreased resting-state FC between the precuneus and the right DLPFC. The DLPFC serves as a canonical node within the executive control network (ECN), essential for higher-order cognitive functions including working memory, planning, goal-directed behavior, and importantly, the top-down regulation of attention and emotion (17, 41–45). Our finding of reduced FC between the precuneus and the DLPFC points specifically to impaired interplay between the DMN and ECN. This DMN-ECN decoupling or antagonism is increasingly recognized as a feature of various stress-related and psychiatric conditions, reflecting difficulties in balancing internally focused thought with external task demands (37, 43). This breakdown in communication between self-referential processing (precuneus) and executive control (DLPFC) likely has significant functional consequences. It may reflect the neurotoxic effects of chronic stress on prefrontal circuits, impairing their ability to modulate DMN activity (9, 42). Functionally, this could manifest as difficulty disengaging from negative internal states (e.g., rumination contributing to exhaustion) or flexibly allocating attentional resources, potentially underlying the subjective cognitive complaints and objective executive function deficits reported in burnout and exhaustion disorder (1, 46–48). Underscoring the clinical relevance of this FC disruption, we found that weaker FC of the precuneus-DLPFC was significantly associated with higher levels of EE. This provides direct evidence linking the impaired functional dialogue between these key networks to the severity of this cardinal burnout symptom, suggesting that compromised executive modulation of internal states is a key factor in the experience of feeling emotionally depleted. This specific finding, linking weakened FC of the precuneus-DLPFC to EE, carries important implications for potential therapeutic interventions. The DLPFC is an accessible and well-established target for non-invasive brain stimulation techniques. Modulating DLPFC activity and its network connectivity using methods such as transcranial magnetic stimulation (TMS), or transcranial direct current stimulation (tDCS) has shown promise for treating conditions involving emotional dysregulation and cognitive deficits, such as depression (49). Our results suggest that interventions aimed at enhancing DLPFC function or specifically strengthening its coupling with the DMN (precuneus) might offer a novel avenue for alleviating emotional exhaustion in burnout.

Extending beyond functional alterations, our imaging transcriptomics analysis provided initial insights into the potential molecular underpinnings of burnout by linking the observed fALFF changes to the spatial expression patterns of genes significantly enriched in key biological domains. Specifically, these enrichments highlight pathways crucial for neuronal function, including neuronal excitability [e.g., membrane depolarization, voltage-gated and potassium ion channels (50)] and synaptic organization, suggesting that altered fALFF may reflect underlying molecular profiles affecting neuronal firing efficiency and synaptic integrity, potentially contributing to burnout symptoms. Furthermore, the significant enrichment of the JNK stress response pathway (51) provides a molecular link between chronic stress exposure and cellular adaptation within affected brain regions, where sustained activation is known to promote maladaptive processes such as neuroinflammation and synaptic dysfunction (52), potentially driving the observed fALFF alterations. Intriguingly, the enrichment in pathways for immune modulation, specifically the negative regulation of cytokine signaling, suggests complex neuroimmune dynamics (53, 54). While peripheral inflammation is often noted in burnout and chronic stress states (6, 55–57), this central finding could represent a compensatory attempt by affected brain regions to dampen local neuroinflammation (52) or reflect altered central immune responsiveness distinct from systemic patterns, perhaps related to chronic Hypothalamic–Pituitary–Adrenal (HPA) axis or immune cell exhaustion (53, 54). These pathways are likely interconnected, with stress signaling, neuronal activity, and immune responses influencing one another. It is crucial to acknowledge the correlational nature of imaging transcriptomics, which associates functional changes with normative gene expression atlases rather than direct measurements in participants. Consequently, further validation is needed. Nevertheless, these findings collectively suggest that burnout-related functional brain alterations are spatially associated with gene expression patterns reflecting potentially compromised neuronal function, heightened cellular stress responses, and complex neuroimmune adjustments. This integrated perspective offers novel insights into the potential molecular pathophysiology of burnout, highlighting specific pathways for future mechanistic investigation and potential therapeutic targeting.

Complementing the transcriptomic findings, the spatial correlation between the altered fALFF patterns and the distribution of multiple neurotransmitter systems provides further mechanistic insights. Specifically, we observed correlations with receptors/transporters integral to the serotonin (5HT1a, 1b, 2a, SERT), norepinephrine (NAT), GABA (GABAa), glutamate (mGluR5), and endocannabinoid (CB1) systems. The convergence on these specific systems is highly relevant to burnout pathophysiology. The correlation with serotonin targets (5-HT receptors, SERT) resonates with the known role of serotonin in regulating mood, anxiety, stress coping, sleep, and cognitive functions-domains profoundly affected in burnout (58, 59). Similarly, the association with the norepinephrine transporter (NAT) points towards alterations in noradrenergic pathways, which are central to arousal, vigilance, and the physiological stress response (60–62). The implication of the GABAergic system (GABAa) suggests potential disruption in inhibitory control, relevant to anxiety and stress coping (63–65), while the link to the metabotropic glutamate receptor mGluR5 highlights the involvement of excitatory glutamate signaling, a system profoundly sensitive to stress and crucial for synaptic plasticity (66–68). The spatial correlation with the CB1 receptor distribution in the endocannabinoid system is particularly compelling. CB1 receptors are highly abundant in brain regions critical for emotional processing, stress adaptation, and cognitive function, including the prefrontal cortex, hippocampus, and amygdala (69–71). Furthermore, the endocannabinoid system interacts extensively with the other neurotransmitter systems identified in our analysis (serotonin, norepinephrine, GABA, glutamate), suggesting it may function as a central node integrating the molecular impact of chronic stress (69). The spatial covariance between burnout-related fALFF changes and normative CB1 expression therefore suggests that impaired endocannabinoid modulation, potentially stemming from chronic stress exposure in the pathophysiology of burnout, contributing to altered neuronal activity patterns and associated symptoms.

Several limitations of this study warrant consideration when interpreting our findings. First, the cross-sectional design precludes definitive conclusions regarding causality. While we observed significant associations between burnout, altered brain activity/FC, and molecular markers, we cannot ascertain whether these neurobiological changes are a cause or consequence of chronic occupational stress and burnout. Longitudinal studies are needed to track the temporal dynamics of these alterations and establish causal relationships. Second, a significant limitation of our study is that our sample consisted exclusively of female nurses. While this homogeneity allowed for a focused investigation within a highly relevant and at-risk population, it precludes any analysis of potential sex differences and limits the generalizability of our findings. This is a particularly important consideration, as emerging evidence suggests that the prevalence, clinical presentation, and societal risk factors for burnout can differ between men and women (72, 73). Therefore, it remains unclear whether the neural correlates of burnout identified in our study are specific to female nurses or represent a more universal mechanism. We strongly advocate for future large-scale studies with balanced sex representation to explicitly test for sex differences and determine the extent to which these neurobiological findings are consistent across genders and occupational groups. Third, while the AHBA provides invaluable spatial transcriptomic data, it is a post-mortem atlas derived from a limited number of individuals. Therefore, our molecular findings are correlational and provide indirect evidence for the *in vivo* molecular changes occurring in nurses with burnout. Future studies employing in vivo molecular imaging techniques or peripheral biomarkers would be beneficial to validate and extend these transcriptomic insights. Finally, although we controlled for several potential confounding factors (age, education, TIV, anxiety, depression), residual confounding cannot be entirely ruled out. Factors such as specific workplace stressors, individual coping mechanisms, lifestyle factors (e.g., sleep quality, diet, exercise) and co-occurring subclinical mental or physical health conditions could potentially influence the observed neuroimaging and molecular alterations.

## Conclusion

In conclusion, this multi-level study provides compelling evidence for specific neurobiological correlates of occupational burnout in female nurses. We demonstrated that burnout is associated with decreased local spontaneous activity in the precuneus, and weakened FC between the precuneus and right DLPFC, and that these neuroimaging alterations are clinically relevant, correlating with core burnout symptoms, particularly EE. Furthermore, our imaging transcriptomics analysis and neurotransmitter correlation findings offer initial insights into potential molecular mechanisms underlying these functional disruptions, suggesting alterations in neuronal excitability, synaptic organization, stress response pathways, neuroinflammation, and multiple neurotransmitter systems, particularly the serotonergic system. These converging findings advance our understanding of burnout as a biologically grounded condition characterized by specific disruptions in brain function and underlying molecular alterations. Importantly, the identified precuneus-DLPFC connectivity deficit suggests potential therapeutic targets for interventions aimed at alleviating burnout symptoms. Non-invasive brain stimulation techniques targeting the DLPFC, as well as cognitive and behavioral therapies that engage executive functions, may hold promise for restoring healthier brain network dynamics and mitigating the debilitating effects of chronic occupational stress. Future research should build upon these findings by employing longitudinal designs, expanding to more diverse populations, utilizing *in vivo* molecular techniques, and exploring broader brain and molecular landscapes to further elucidate the complex neurobiological underpinnings of burnout and develop more targeted and effective interventions.

## Data Availability

The original contributions presented in the study are included in the article/Supplementary material, further inquiries can be directed to the corresponding authors.

## Footnotes

^1^ http://geneontology.org/

^2^ https://github.com/murraylab/brainsmash

^3^ https://github.com/juryxy/JuSpace
