# Supplementary material for: Transcriptional and neurotransmitter signatures of cerebral spontaneous neural activity in nurses with burnout
Source: Front Public Health. 2025 Aug 21;13:1630294. doi: 10.3389/fpubh.2025.1630294 (PMC12408554; doi:10.3389/fpubh.2025.1630294)
Supplement: Supplementary file 1 [file Supplementary_file_1.docx]

Supplementary Material

# **1 Detailed fMRI parameters**

Functional images were acquired using an echo-planar imaging (EPI) sequence with the following parameters: repetition time (TR) = 3000 ms, echo time (TE) = 35 ms, field of view (FOV) = 240 mm × 240 mm, slice thickness = 5 mm, voxel size = 3.75 × 3.75 × 4 mm, number of volumes = 128. High-resolution T1-weighted anatomical images were also acquired using a magnetization-prepared rapid gradient-echo (MPRAGE) sequence with the following parameters: TR = 7.5 ms, TE = 2.8 ms, FOV = 240 mm × 240 mm, slice thickness = 1.0 mm, number of slices = 152, flip angle = 15°, and voxel size = 0.5 × 0.5 × 1 mm.

# **2 Detailed preprocessing procedures**

Preprocessing of the fMRI data was performed using the Data Processing Assistant for Resting-State fMRI (DPARSF) toolbox (1), based on SPM12 (Statistical Parametric Mapping). The preprocessing steps included: (1) removal of the first 10 volumes to allow for signal equilibration; (2) slice timing correction; (3) realignment to the middle volume to correct for head motion; (4) exclusion of participants with maximum displacement > 3 mm or rotation > 3°; (5) spatial normalization to the standard Montreal Neurological Institute (MNI) space achieved through the DARTEL (Diffeomorphic Anatomical Registration Through Exponentiated Lie Algebra) alignment method and resampling to 3 × 3 × 3 mm3; (6) spatial smoothing with a Gaussian kernel of 4 mm full-width at half-maximum (FWHM); (7) linear regression to reduce errors; (8) regression of nuisance covariates; Filtering is necessary for the computation of FC.

Total intracranial volume (TIV) was obtained for all participants through the Computational Anatomy Toolbox (CAT12) in SPM12, which involved bias field correction, skull stripping, alignment to the MNI template, and segmentation into gray matter, white matter, and cerebrospinal fluid.

Table S1 Correlations between functional alterations and clinical scales in the burnout group

| **Functional alterations** | **Clinical scales** | ***r*** | ***P*** |
| --- | --- | --- | --- |
| fALFF of precuneus | EE | -0.290 | *0.039* |
|  | DP | -0.312 | *0.026* |
|  | PA | 0.287 | *0.041* |
| FC between precuneus and right DLPFC | EE | -0.308 | *0.028* |
|  | DP | 0.084 | 0.560 |
|  | PA | 0.011 | 0.937 |

Within the burnout group, fALFF values in the precuneus were negatively correlated with both EE and DP, while showing a positive correlation with PA. Furthermore, the FC between the precuneus and the right DLPFC was also negatively correlated with EE scores.

Italic represents a significant correlation between functional alterations and clinical scales.

**Abbreviations:** fALFF, fractional amplitude of low-frequency fluctuations; EE, emotional exhaustion; DP, depersonalization; PA, personal accomplishment; FC, functional connectivity; DLPFC, dorsolateral prefrontal cortex.

# **Table S2 Results of spatial correlation analyses between fALFF alterations and neurotransmitter distribution**

| **Receptor/Transporter** | **Map** | **Pearson *r*** | **Exact *P*-value** |
| --- | --- | --- | --- |
| 5HT1a | 5HT1a_WAY_HC36 | -0.3197 | 0.0234^*^ |
|  | 5HT1a_cumi_hc8_beliveau | -0.6051 | 0.0004^*^ |
| 5HT1b | 5HT1b_P943_HC22 | 0.6494 | 0.0002^*^ |
|  | 5HT1b_az_hc36_beliveau | 0.2946 | 0.0072^*^ |
| 5HT2a | 5HT2a_ALT_HC19 | 0.3290 | 0.0008^*^ |
|  | 5HT2a_cimbi_hc29_beliveau | 0.1634 | 0.3083 |
| 5HT4 | 5HT4_sb20_hc59_beliveau | 0.0931 | 0.3875 |
| CB1 | CB1_FMPEPd2_hc22_laurikainen | 0.5488 | 0.0002^*^ |
| D1 | D1_SCH23390_c11 | 0.1263 | 0.2364 |
| D2 | D2_RACLOPRIDE_c11 | 0.2033 | 0.0524 |
|  | D2_fallypride_hc49_jaworska | 0.0747 | 0.5019 |
| DAT | DAT_DATSPECT | -0.0257 | 0.8164 |
| FDOPA | FDOPA_f18 | 0.1209 | 0.2596 |
| GABAa | GABAa_FLUMAZENIL_c11 | 0.2946 | 0.0446^*^ |
|  | GABAa_flumazenil_hc16_norgaard | 0.4552 | 0.0170^*^ |
| KappaOp | KappaOp_LY2795050_hc10_ShokriKojori | 0.1559 | 0.3013 |
| MU | MU_CARFENTANIL_c11 | -0.2599 | 0.2696 |
|  | MU_carfentanil_hc39_turtonen | -0.1829 | 0.4539 |
| NAT | NAT_MRB_c11 | -0.2549 | 0.0158^*^ |
| NMDA | NMDA_ge179_29hc_galovic2021 | 0.0110 | 0.9428 |
| SERT | SERT_DASB_HC30 | -0.1890 | 0.0768 |
|  | SERT_MADAM_c11 | -0.1726 | 0.1086 |
|  | SERT_dasb_hc100_beliveau | -0.2162 | 0.0406^*^ |
| VAChT | VAChT_feobv_hc18_aghourian | 0.0596 | 0.5773 |
|  | VAChT_feobv_hc4_tuominen | 0.0593 | 0.5729 |
|  | VAChT_feobv_hc5_bedard | 0.0529 | 0.6301 |
| mGluR5 | mGluR5_abp_hc22_rosaneto | 0.4650 | 0.0022^*^ |
|  | mGluR5_abp_hc28_dubois | 0.3386 | 0.0266^*^ |
|  | mGluR5_abp_hc73_smart | 0.3100 | 0.0042^*^ |

Spatial correlation between fALFF alterations and neurotransmitter distribution; ^*^ *P* < 0.05.

**Abbreviations:** 5HT1a, serotonin 5-hydroxytryptamine receptor subtype 1a; 5HT1b, serotonin 5-hydroxytryptamine receptor subtype 1b; 5HT2a, serotonin 5-hydroxytryptamine receptor subtype 2a; 5HT4, serotonin 5-hydroxytryptamine receptor subtype 4; CB1, cannabinoid 1 receptor; CBF, cerebral blood flow; D1, dopamine D1 receptor; D2, dopamine D2 receptor; DAT, dopamine transporter; FDOPA, 6-fluoro-(18F)-L-3,4-dihydroxyphenylalanine; GABAa, gamma-aminobutyric acid type a; MU, μ-opioid receptor; NAT, noradrenaline transporter; NMDA, N-methyl-D-aspartic acid receptor; SERT, serotonin transporter; VAChT, vesicular acetylcholine transporter; mGluR5, metabotropic glutamate receptor 5.

**References:**

1. Chao-Gan Y, Yu-Feng Z. DPARSF: A MATLAB Toolbox for "Pipeline" Data Analysis of Resting-State fMRI. *Front Syst Neurosci*. (2010) 4:13. doi:10.3389/fnsys.2010.00013
